# Influence of age on static postural control in adults with type 2 diabetes mellitus: a cross-sectional study

**DOI:** 10.3389/fendo.2023.1242700

**Published:** 2023-09-19

**Authors:** Yujun Zhuang, Zhenzhen Hong, Lijuan Wu, Chunyan Zou, Yan Zheng, Liming Chen, Lianhua Yin, Jiawei Qin

**Affiliations:** ^1^ Department of Outpatient, Quanzhou First Hospital Affiliated to Fujian Medical University, Quanzhou, China; ^2^ Department of Endocrinology, Quanzhou First Hospital Affiliated to Fujian Medical University, Quanzhou, China; ^3^ Health Management Center, The Second Affiliated Hospital of Fujian University of Traditional Chinese Medicine, Fuzhou, China; ^4^ Department of Rehabilitation Medicine, Quanzhou First Hospital Affiliated to Fujian Medical University, Quanzhou, China

**Keywords:** type 2 diabetes mellitus, age, center of pressure (COP), static postural control, cross-sectional

## Abstract

**Aim:**

It was the aim of this study to assess static postural control characteristics in people with type 2 diabetes mellitus (T2D) of different ages using a force platform. A relationship was also established between static postural control parameters and age in this study.

**Methods:**

A total of 706 participants with T2D were included in this study. The participants were stratified into three age groups: Group 1 (<60 years old), Group 2 (60–70 years old), and Group 3 (>70 years old). Static postural control assessment during two-leg stance was performed on a force platform by all participants. The center of pressure (CoP)-related parameters were measured under two stance conditions (eyes open and closed). Kruskal–Wallis tests were applied to explore the difference among the different age groups. Multivariate regression analysis was performed to determine the relation between age and static postural control parameters.

**Results:**

Group 1 (<60 years old) had significantly less CoP total tracking length (TTL), sway area (SA), and CoP velocity along the *Y* direction (V-Y) under both eyes-open and eyes-closed conditions compared with Group 2 (60–70 years old) and Group 3 (>70 years old). Group 1 (<60 years old) had significantly less CoP maximum sway length along the *X* direction (MSL_X) and longer tracking length each area unit (TTL/SA) under the eyes-open condition compared with Group 2 (60–70 years old) and Group 3 (>70 years old). There was a significantly positive correlation between age and the most static postural parameters such as CoP TTL, SA, MSL-X, MSL-Y, and V-Y. There was a significantly negative correlation between age and TTL/SA.

**Conclusion:**

This study suggested that older T2D participants had worse static postural control ability than younger ones. Most static postural parameters presented a significant correlation with age; the higher the age, the worse the static postural control.

## Introduction

According to the IDF (International Diabetes Federation) Diabetes Atlas reports, the global diabetes prevalence was estimated to be 10.5% in 2021, which is over 536 million people, and increasing with age (1). The diabetes prevalence globally was expected to increase to 12.2% (783.2 million people) in 2045, and over 90% of all diabetes was type 2 diabetes (T2D) (1). China was the country with the largest number of people with diabetes aged 20–79 years in 2021 (1).

As a primary cause of both fatal and non-fatal injuries in the elderly, falls appeared to be a growing and costly public health challenge (2). Fall injuries limited individuals’ activities of daily life and social engagements, which resulted in further physical capacity decline, social isolation, and feelings of helplessness. In 2015, the total cost of fatal fall was $637.2 million, and the treatment cost of non-fatal fall was $31.3 billion; the average medical treatment cost of a fall was up to $9,780 in America (3). Compared with healthy adults of similar age, the patients with T2D were at high risk of falling (4). As one of the mostly identified risk factors, postural control impairment was recognized to increase the risk of falls and fracture incidence in the patients with diabetes (5).

Clinical tests were usually used to evaluate the postural control ability, such as Time Up and Go (TUG), Berg Balance Scale (BBS), and One Leg Standing Test (OLST). However, these clinical tests were not sensitive enough to evaluate the postural control ability (6). Laboratory testing collecting CoP data from the force platform was a quantitative assessment for postural control (7). For patients with diabetes, the decline of static posture control ability is one of the common complications (8). A previous cross-sectional study found that T2D participants had more falls than healthy controls, and increased static postural instability was associated with the falls (9).

Static posture control refers to the ability to maintain the center of pressure (CoP) within the base of support (10). The CoP displacements derived from the force platform were considered the most reliable approach to assess the static postural balance control (11). The efficiency of multiple systems including the visual, vestibular, and proprioception system was important for the subject to maintain postural balance. Age is a crucial factor that affects the physiological and metabolic functions of the human body, including the function of the nervous system (12). Age-related physiological changes were expected to cause postural balance impairment and higher fall risk (13). A previous study found that older adults demonstrated larger CoP swaying areas under both eyes-open and eyes-closed conditions compared with younger individuals (14). A greater CoP sway velocity was found in the older adults who had fallen at least once in the last year compared with the non-fallers (15).

Previous studies have found that postural balance of healthy adults deteriorated with aging, and the postural balance measures were significantly associated with age (16, 17). Our previous research found that T2D patients had poor static postural balance compared with the control group (18). It is important to understand the influence of age on the static posture control of T2D patients. Firstly, it can help doctors and patients become aware of this impact and take measures to prevent or reduce aggravation. Secondly, doctors can be guided to develop rehabilitation plans for T2D patients of different age groups, helping them improve their static posture control ability and functional activities. Finally, although the influence of research age on static posture control has been confirmed, a more in-depth research in this field is needed, especially for diabetes management strategies for T2D patients of different ages. Therefore, the objective of this study was to explore the influence of age on the static posture control in T2D patients.

## Methods

### Participants

A total of 706 T2D participants were included in the study. All participants were recruited from the health examination department of Fujian Province Second People’s Hospital from January 2015 to June 2020. Potential participants were contacted and encouraged to complete relevant questionnaires to identify their eligibility for this study. The relevant questionnaire contained the research introduction, inclusion criteria, exclusion criteria, and static postural control assessment process.

The inclusion criteria were as follows: adults who were diagnosed as having T2D by the clinician and voluntarily participation in this study. The exclusion criteria were as follows (1): blindness or deafness; (2) symptoms and signs related to rheumatic disease, osteoarthritis, cardiovascular disorders, and neuromuscular disease that affect postural balance; (3) dementia or malignancy; (4) history of spine or lower limb surgery; and (5) lower limb-related injuries in the past 12 months. This research was approved by the Fujian Province Second People’s Hospital Ethics Committee (approval number SPHFJP-K2019059-02).

### Assessment

Demographic and clinical information, such as age, gender, body mass index (BMI), systolic and diastolic blood pressure, smoking, heart rate, and FBG (fasting blood glucose), were collected in the initial recruitment period.

A certified and experienced physiotherapist evaluated the participants’ static postural balance function using a force platform (Super Balance, Bismarck, Germany). These measurements were taken in a separate, quiet, and bright room. The participants were told to remove their shoes and stand on the firm force platform with their feet hip-width and arms at their sides. The participants maintained their body upright, and kept a stable CoP as possible, with the static postural control evaluation phases including the following: firstly, 30 s of eyes-open standing on the force platform, followed by 30 s of eyes-closed standing following a 1-minbreak. Each trial was only completed once for each participant, and they were all evaluated in this sequence. With eyes open, participants faced forward; with eyes closed, they voluntarily closed their eyes. All of the participants’ sessions took place in the morning. With eyes open and closed, the CoP’s change track (posture diagram) was captured. If the participants made their steps, or grabbed the handrails, the evaluation would be interrupted and restarted. To protect the participants, the assessor stood next to them.

The following parameters were collected: (1) total tracking length (TTL, mm) refers to the distance traveled by CoP in a given time; the overall CoP tracking length shows the amount of spontaneous body shaking; (2) sway area (SA, mm^2^) is the size of the CoP trajectory map and is negatively related to the postural control ability, which could be used to assess overall postural balance; (3) tracking length each area unit (TTL/SA, mm) is the quotient of TTL divided by SA, which also reflects the postural stability; (4) CoP maximum sway length on the *X* axis (MSL-X, mm) and *Y* axis (MSL-Y, mm) corresponds to the CoP longest shaking distance along the medial–lateral and anterior–posterior directions, respectively; (5) CoP velocity on the *X* axis (V-X, mm/s) and *Y* axis (V-Y, mm/s) refer to the speed of CoP shaking along the medial–lateral and anterior–posterior directions, respectively; (6) Romberg quotient (RQ) refers to the tracking map area ratio with eyes closed and open (19), which shows the compensatory strategy ability of vestibular function and proprioception in posture control with the absence of visual factors, reflecting the effect of visual feedback in posture control (18–21).

### Statistical analysis

Statistical Package for the Social Sciences (SPSS, version 24.0) was used for statistical analysis. Kolmogorov–Smirnov test was used to assess the normality distribution of the variables. The normally distributed continuous variables were presented as means and standard deviation (SD), non-normally distributed variables were presented as median and interquartile range (IQR), and categorical variables were presented as frequencies or percentages.

One-way analysis of variance (ANOVA) or Kruskal–Wallis tests were used to determine the difference among the different age groups, and *post-hoc* pairwise comparisons were adopted. Chi-square analysis was used for categorial data. Multivariate regression analysis was performed to explore the relation of age and static postural control parameters while adjusting for hypertension, current smoking status, and heart rate. *p* < 0.05 was considered statistically significant.

## Results

A total of 706 participants were divided into different age groups: Group 1 (<60 years old, *n* = 233), Group 2 (60–70 years old, *n* = 287), and Group 3 (>70 years old, *n* = 186). **Table 1** demonstrates the characteristics of the participants for three groups. No significant differences were found for gender, BMI, BMI category, diastolic pressure, and fasting blood glucose in the three groups (*p* > 0.05). Significant differences were displayed in age, systolic pressure, number of hypertension, current smoking status, and heart rate among the three groups (*p* < 0.05).

**Table 1:** Characteristics of participants.

	Group 1 (*n* = 233) <60 years	Group 2 (*n* = 287) 60–70 years	Group 3 (*n* = 186) >70 years	*p*
Age, median (IQR), years	53.00 (7.00)	65.00 (6.00)	74.00 (3.00)	<0.001
Gender, male/female	152/81	188/99	114/72	0.605
BMI, median (IQR), kg/m^2^	24.47 (3.29)	24.33 (3.78)	24.40 (3.95)	0.685
BMI category, *n*				0.943
1 Normal weight	98	129	81	
2 Overweight	107	123	80	
3 Obesity	28	35	25	
Systolic pressure, median (IQR), mmHg	128.00 (22.00)	135.00 (20.00)	143.00 (20.00)	<0.001
Diastolic pressure, median (IQR), mmHg	79.00 (13.00)	80.00 (14.00)	78.00 (16.00)	0.102
Hypertension, *n*	76	180	138	<0.001
Current smoking, *n*	44	35	12	0.001
Heart rate, median (IQR), bpm	78.00 (13.00)	75.00 (12.00)	74.00 (17.00)	0.026
FBG, median (IQR), mmol/L	7.22 (2.27)	6.82 (2.22)	6.97 (2.15)	0.193

IQR, interquartile range; BMI, body mass index; FBG, fasting blood glucose; BMI category: normal weight (BMI = 18.5–23.9), overweight (BMI = 24–27.9), and obesity (BMI ≥ 28).

**Table 2** demonstrates the static postural balance differences across the three groups. For the static postural balance measurements with eyes open, there were statistically significant differences in TTL, SA, TTL/SA, MSL-X, MSL-Y, and V-Y in all groups. *Post-hoc* pairwise comparison results found that Group 1 had significantly less TTL, SA, MSL-X, and V-Y compared to Group 2 and Group 3; Group 1 showed significantly less MSL-Y compared to Group 3; Group 1 had significantly more TTL/SA compared to the other two groups. For the static postural balance measurements with eyes closed, there were statistically significant differences in TTL, SA, MSL-X, MSL-Y, and V-Y in all groups. *Post-hoc* pairwise comparison results found that Group 1 had significantly less TTL, SA, MSL-X, MSL-Y, and V-Y compared to Group 3; Group 1 had significantly less V-Y compared to Group 2; and Group 2 had significantly less MSL-X compared to Group 3. Regarding RQ, no significant difference was found in the three groups.

**Table 2:** Static postural balance parameters across the three groups.

		Group 1 (*n* = 233) <60 years	Group 2 (*n* = 287) 60–70 years	Group 3 (*n* = 186) >70 years	*p*	*Post-hoc* pairwise comparisons, *p*		
						1 vs. 2	1 vs. 3	2 vs. 3
Eyes-open	TTL	182.51 (116.12)	206.60 (98.69)	213.76 (93.38)	<0.001	0.018	<0.001	0.36
	SA	113.92 (119.54)	143.52 (120.71)	162.10 (136.87)	<0.001	0.001	<0.001	0.217
	TTL/SA	1.60 (1.23)	1.36 (1.00)	1.35 (0.89)	0.001	0.016	0.01	0.87
	MSL-X	10.34 (6.58)	12.77 (7.12)	13.39 (8.00)	<0.001	<0.001	<0.001	0.315
	MSL-Y	16.81 (8.11)	17.83 (8.35)	18.73 (8.06)	0.004	0.065	0.004	0.724
	V-X	3.40 (2.80)	3.60 (2.10)	3.65 (2.10)	0.134	/	/	/
	V-Y	4.40 (2.50)	4.90 (2.50)	5.35 (2.20)	<0.001	0.002	<0.001	0.253
Eyes-closed	TTL	266.61 (127.35)	282.39 (157.37)	309.60 (181.45)	0.002	0.137	0.001	0.195
	SA	162.88 (160.79)	195.85 (203.03)	222.41 (244.22)	<0.001	0.06	<0.001	0.125
	TTL/SA	1.51 (1.17)	1.50 (1.03)	1.40 (0.98)	0.096	/	/	/
	MSL-X	11.81 (7.55)	12.80 (7.55)	14.03 (9.57)	<0.001	0.096	<0.001	0.048
	MSL-Y	22.08 (9.66)	23.94 (11.89)	25.16 (11.35)	0.024	0.135	0.028	1
	V-X	4.20 (2.95)	4.00 (3.10)	4.40 (3.15)	0.12	/	/	/
	V-Y	7.10 (3.35)	7.70 (4.00)	8.40 (4.70)	<0.001	0.019	<0.001	0.3
Romberg quotient		156.60 (156.85)	142.00 (124.40)	138.40 (153.70)	0.365	/	/	/

TTL, total tracking length; SA, sway area; TTL/SA, tracking length each area unit; MSL-X, CoP maximum sway length on the X axis; MSL-Y, CoP maximum sway length on the Y axis; V-X, CoP velocity on the X axis; V-Y, CoP velocity on the Y axis; 1 vs. 2, Group 1 vs. Group 2; 1 vs. 3, Group 1 vs. Group 3; 2 vs. 3, Group 2 vs. Group 3.

**Table 3** demonstrates the multivariate regression analysis results between age and static postural balance parameters when adjusting for hypertension status, current smoking status, and heart rate. We found a significantly positive relationship between age and the most static postural balance measurements (TTL, SA, MSL-X, MSL-Y, and V-Y) with eyes open. We also found a significantly positive relationship between age and most static postural balance measurements (TTL, SA, MSL-X, MSL-Y, V-X, and V-Y) under the eyes-closed condition. A significantly negative relationship between age and TTL/SA was found with eyes open and closed. No significant difference was found between age and RQ.

**Table 3:** Multivariate regression analysis for the relationship of age and static postural control parameters.

		Age (adjusted for hypertension, smoking status, and heart rate)		
		Beta	*t*	*p*
Eyes-open	TTL	0.113	2.788	0.005
	SA	0.161	4.023	<0.001
	TTL/SA	−0.084	−2.059	0.04
	MSL-X	0.197	4.923	<0.001
	MSL-Y	0.113	2.811	0.005
	V-X	0.043	1.044	0.297
	V-Y	0.157	3.896	<0.001
Eyes-closed	TTL	0.171	4.249	<0.001
	SA	0.16	3.962	<0.001
	TTL/SA	−0.13	−3.193	0.001
	MSL-X	0.191	4.74	<0.001
	MSL-Y	0.096	2.362	0.018
	V-X	0.107	2.634	0.009
	V-Y	0.188	4.676	<0.001
Romberg quotient		−0.019	−0.459	0.646

TTL, total tracking length; SA, sway area; TTL/SA, tracking length each area unit; MSL-X, CoP maximum sway length on the X axis; MSL-Y, CoP maximum sway length on the Y axis; V-X, CoP velocity on the X axis; V-Y, CoP velocity on the Y axis.

## Discussion

This study explored the static postural balance control characteristics regarding different age T2D participants and the relationship of age and different postural balance measurements. This study demonstrated that most static postural balance control measurements were poorer with age, and worse in the oldest age group (70–80 years old). Our study confirmed that the longer TTL, MSL-X, and MSL-Y, the bigger the SA, and the faster V-Y with eyes open and closed in the older T2D age group. Moreover, this study found a significant relationship between age and static postural balance measurements for T2D participants; the higher the age, the poorer the balance function.

Previous studies have found that balance function was worse in subjects with T2D compared to healthy controls (22, 23). The reasons that T2D subjects had impaired balance were multifaceted. Since postural balance control required the integration of multiple sensorimotor systems and cognitive process, the diabetes-related and age-related deterioration in sensorimotor systems as well as cognitive function could disrupt the ability of maintaining postural balance (24). Moreover, autonomic damage of the subjects with T2D masked much of the stress of the inability to optimize postural balance, while the older subjects had more severe autonomic damage (22). Diabetic peripheral neuropathy (DPN) is a common complication of T2D that causes impairment of proprioception and balance functions. Vibration perception threshold, age, and muscle strength were important predictors of balance function and falling fear in T2D patients (25). Similarly, Yumin et al. concluded that both T2D patients with and without DPN experienced worse balance, sensation, and mobility when compared to healthy control subjects (26). Significant differences were detected in those with longer diabetes duration and excessive medication use (26). The fact that older T2D adults are at heightened risk for postural balance impairments and falls is not surprising.

Consistent with the findings of this study, a previous study demonstrated that postural stability measured by the Biodex Stability System was significantly related to age, and older age was correlated with worse postural stability (27). Similarly, a previous study found that older adults had more difficulty and may have needed more attention to stand still than young adults (28). Furthermore, dysfunction of motor coordination was also found in T2D patients along with an increasing risk of falling, compared to healthy controls in a similar age group (29). The static postural balance with vision control worsened as the T2D patients’ age increased (29). In addition, Collins et al. suggested the increased heterogeneity of postural control ability in healthy older adults compared with young adults (30). Postural control is achieved by integrating information inputs from the visual, somatosensory, and vestibular systems at the central nervous system level (31). However, the function of visual, somatosensory, and vestibular systems continued to deteriorate due to aging (31, 32). The T2D participants with higher falls risk showed greater anterior–posterior postural sway under the eyes-open condition compared to those T2D participants with lower falls risk (32). The T2D participants with higher falls risk showed greater anterior–posterior and medial–lateral postural sway with eyes closed compared to those T2D participants with lower falls risk (32). Wettasinghe et al. reported that increased postural sway with eyes open and closed was significantly associated with falls (33). Most postural sway parameters in the older age group were significantly higher than those in the relatively younger group from this study. In addition, the older age group showed increased CoP sway compared with the younger age group, and age was significantly positively associated with most CoP sway length and velocity, indicating that increasing age may increase the falls risk in the T2D patients. The significantly negative relation between age and TTL/SA was found in this study; the mechanism may be that T2D individuals triggered a stiffening strategy in the postural task (32), while older T2D individuals performed a worse stiffening strategy. Lee et al. found that older T2D patients had more decrease in reactivity equilibrium control, and these changes may result from muscle weakness and plantar insensitivity (34). These findings combined with our research results suggested that older T2D patients should have higher falls risk.

The influence of age on posture control in T2D patients may be complex, and may vary depending on the patient’s health and disease status. However, generally speaking, the older the patient is, the worse the posture control of diabetes patients may be. This may be due to a decrease in physical function, muscle and joint stiffness, and a decrease in balance and reaction time in elderly individuals, which makes it difficult to maintain the correct posture and balance when standing. In addition, the nervous system of elderly people may also be damaged, which can affect their sensory and coordination abilities, further affecting posture control. The severity of frailty in elderly people with T2D increased with age, and frailty was associated with lower systolic blood pressure, higher triglyceride levels, poorer nutritional status, and less independence in performing instrumental activities of daily life and poorer postural balance (35). Fear of falling was common in T2D patients, and was associated with impaired postural function and increased risk of falls (36, 37). Fear of falling and insufficient balance confidence were highly prevalent, and much more severe in older T2D patients (37). Older age adversely affected foot sensitivity, and decreased foot sensitivity was associated with poorer balance and postural stability (38). Cognitive function may play an important role in postural balance function among different T2D age groups. Blackwood found that executive function was associated with falls after adjusting for demographic and physical mobility and strength-related variables (39). As a well-known risk factor, impaired postural control during quiet stance was common in T2D and correlated well with a higher prevalence of falls (8, 40). Therefore, older patients with T2D may need more active posture control training and rehabilitation exercise to help them maintain correct posture, reduce symptoms, and reduce the risk of falls and other injuries.

The strengths of this study were the number of participants and the quantitative evaluation of the static postural balance. This study had several limitations: firstly, some issues affecting static postural balance were not collected, like diabetes course, diabetes drug use, muscle strength, body composition, and physical activity status. Thus, only two-leg standing on a firm force platform with eyes open and closed was assessed in this study. Moreover, future research could focus on the differences in posture control measures between diabetes and normal controls at different age groups. Finally, this cross-sectional designed study cannot explore the causal effect inference. The finding of this study provides healthcare professionals a deep understanding of the static postural balance control characteristics and targeted interventions to improve static balance function in T2D patients.

## Conclusion

This study suggested that older T2D patients had worse static postural control performance than younger ones. Most static postural control parameters presented a significant correlation with age; the higher the age, the worse the static postural control. Future research with reference to the aforementioned suggestions is needed.

## Data availability statement

The raw data supporting the conclusions of this article will be made available by the authors, without undue reservation.

## Ethics statement

The studies involving humans were approved by the Second Affiliated Hospital of Fujian University of Traditional Chinese Medicine Ethics Committee. The studies were conducted in accordance with the local legislation and institutional requirements. The participants provided their written informed consent to participate in this study.

## Author contributions

YuZ and ZH contributed equally to this work and share first authorship. YuZ, ZH, and LY designed the study. YuZ and ZH wrote the manuscript. LW, CZ, YaZ, and LC selected data. LY and JQ did statistical analysis. LY and JQ reviewed and edited the manuscript. All authors contributed to the article and approved the submitted version.
